# Induction and antiviral activity of ferret myxovirus resistance (Mx) protein 1 against influenza A viruses

**DOI:** 10.1038/s41598-024-63314-2

**Published:** 2024-06-12

**Authors:** Rubaiyea Farrukee, Lara S. U. Schwab, James B. Barnes, Andrew G. Brooks, Sarah L. Londrigan, Gunther Hartmann, Thomas Zillinger, Patrick C. Reading

**Affiliations:** 1 Department of Microbiology and Immunology, The Peter Doherty Institute for Infection and Immunity, The University of Melbourne, 792 Elizabeth St., Victoria, 3000 Australia; 2 Victorian Infectious Diseases Reference Laboratory, WHO Collaborating Centre for Reference and Research on Influenza, The Peter Doherty Institute for Infection and Immunity, 792 Elizabeth St., Victoria, 3000 Australia; 3 Institute of Clinical Chemistry and Clinical Pharmacology, University Hospital Bonn, 53127 Bonn, Germany

**Keywords:** Virology, Innate immunity

## Abstract

Myxovirus resistance (Mx) proteins are products of interferon stimulated genes (ISGs) and Mx proteins of different species have been reported to mediate antiviral activity against a number of viruses, including influenza A viruses (IAV). Ferrets are widely considered to represent the ‘gold standard’ small animal model for studying pathogenesis and immunity to human IAV infections, however little is known regarding the antiviral activity of ferret Mx proteins. Herein, we report induction of ferret (f)Mx1/2 in a ferret lung cell line and in airway tissues from IAV-infected ferrets, noting that fMx1 was induced to higher levels that fMx2 both in vitro and in vivo. Overexpression confirmed cytoplasmic expression of fMx1 as well as its ability to inhibit infection and replication of IAV, noting that this antiviral effect of fMx1was modest when compared to cells overexpressing either human MxA or mouse Mx1. Together, these studies provide the first insights regarding the role of fMx1 in cell innate antiviral immunity to influenza viruses. Understanding similarities and differences in the antiviral activities of human and ferret ISGs provides critical context for evaluating results when studying human IAV infections in the ferret model.

## Introduction

Following viral infection, host cells deploy a wide range of mechanisms to limit viral infection and spread. As first line of defence, highly conserved cell -associated innate immune sensing receptors such as the cytoplasmic helicase RIG-I can detect viruses, specifically viral nucleic acids, triggering a signalling cascade that results in the rapid induction of diverse antiviral effectors. In our previous work we have demonstrated that the activation of those pathways with synthetic RNA ligands of RIG-I provides potent protection from viral infection with influenza A virus (IAV), severe acute respiratory syndrome coronavirus 2 (SARS-CoV-2) and respiratory syncytial virus (RSV) in mice and ferrets^1–4^. Cell-intrinsic innate antiviral defences include a range of intracellular proteins with antiviral activity that are regulated independently of interferons (IFNs), as well as IFN stimulating genes (ISGs). Collectively, intracellular proteins which mediate antiviral activity against one or more viruses are known as host restriction factors (hereafter referred to as restriction factors). A number of restriction factors have been shown to inhibit IAV at different stages of the virus replication cycle, including during virus entry and uncoating (e.g. IFN-inducible transmembrane (IFITM) proteins), viral genomic replication (e.g. myxovirus resistance (Mx) proteins) and assembly and budding (e.g. tetherin) (reviewed in^5^).

Mx family proteins are IFN-inducible dynamin-like large GTPases that have been reported to mediate antiviral activity against particular RNA and DNA viruses. *Mx* genes are highly conserved within vertebrates which can have one to seven *Mx* gene copies with most mammals express two *Mx* genes, including humans and mice^6^. It is well established that particular Mx proteins from a number of species can act as restriction factors for IAV, including human (h)MxA, but not hMxB, and mouse (m)Mx1, but not mMx2 (reviewed in^7^). hMxA is a cytoplasmic protein that acts to retain IAV nucleocapsids in the cytoplasm, preventing their nuclear import and thereby blocking early steps in the viral replication cycle^8^. In contrast, mMx1 localises to the nucleus and mediates anti-IAV activity by inhibiting primary transcription, possibly by disrupting interactions between the viral nucleoprotein (NP) and PB2 in the ribonucleoprotein complex^9,10^. In fact, mutations in mMx1 that result in redistribution to the cytoplasm abolish its antiviral activity against IAV^11^.

Although less well studied, Mx proteins from other species have also been reported to inhibit members of particular virus families, including rat Mx2 (*Rhabdoviridae* and *Bunyaviridae*), chicken Mx (*Rhabdoviridae* and *Paramyxoviridae*), cow Mx1 and Mx2 (*Rhabdoviridae*) and dog Mx1 and Mx2 (*Rhabdoviridae*) (reviewed in^6^). Aside from hMxA and mMx1, additional proteins reported to mediate anti-IAV activity include nuclear Mx proteins such as rat Mx1^12^, as well as cytoplasmic Mx proteins such as pig Mx1^13^. While ferrets are considered to be the gold standard small animal model to study pathogenesis and immunity to human IAV (reviewed in^14,15^), little is currently known regarding the characteristics and the antiviral activity of host cell restriction factors in ferrets, including the Mx proteins. Herein, we investigated ferret Mx proteins, including their induction in response to type I IFNs, RIG-I agonists and IAV infection in vitro, as well as induction in the airways following IAV-infection of ferrets. Moreover, we determined the major isoform of ferret Mx1 protein induced and used an inducible overexpression system to assess its antiviral activity against IAV.

## Results

### Predicted ferret Mx proteins

*Mx* genes are highly conserved in vertebrates with most mammals expressing both Mx1-like and Mx2-like lineages. Previous studies performed whole genome sequencing on an individual female ferret, as well as RNA-seq on 24 samples from male and female ferrets, allowing for annotation of 19,910 protein-coding sequences^16^ deposited in Genebank in 2015. The annotated sequence (NCBI Accession AEYP00000000) included identification of 3 genes predicted to encode Mx-like proteins, namely fMx1 (with two isoforms, fMx1.1 (NCBI XM_013061287.1) and fMx1.2 (NCBI XM_004762192.2) and fMx2 (NCBI XM_013061286.1). The fMx1.1 mRNA predicted transcript (2,604 base pairs (bp)) contained an additional 23 bp at the 5′ end, as well as an additional 36 bp from residue 248, which are missing from the fMx1.2 predicted transcript (2,545 bp), resulting in an extra 59 nucleotides that are expressed only in fMx1.1. From these genes, the predicted coding sequence is 2,013 bp for fMx1.1 and 1,977 bp for fMx1.2, corresponding to 670 and 658 amino acid sequences, respectively. For fMx2, the gene sequence is 3973 bp, with a predicted coding sequence of 2094 bp and a protein of 697 amino acids.

Annotation of fMx genes was updated in 2020 using data from another study (Bioproject PRJNA776022), with 5 predicted transcript variants of fMx1 (X1-5) and 14 predicted transcript variants of fMx2 (X1-14) added such that the original fMx1.1 and fMx1.2 have been designated variant X3 and X4, respectively, and the original fMx2 showing 99.9% identity to X6 (both 697 residues but with Ala to Thr substitution at residue 246 in X6). Given that short base pair reads (150 bp pair end) were used to determine predicted transcript variants, it is not known which transcript variants represent ‘true’ Mx isoforms that would be induced in biological settings.

### Induction of ferret Mx1 and Mx2 in ferret lung cells in response to ferret IFN-α or RIG-I stimulation

**Figure 1:**
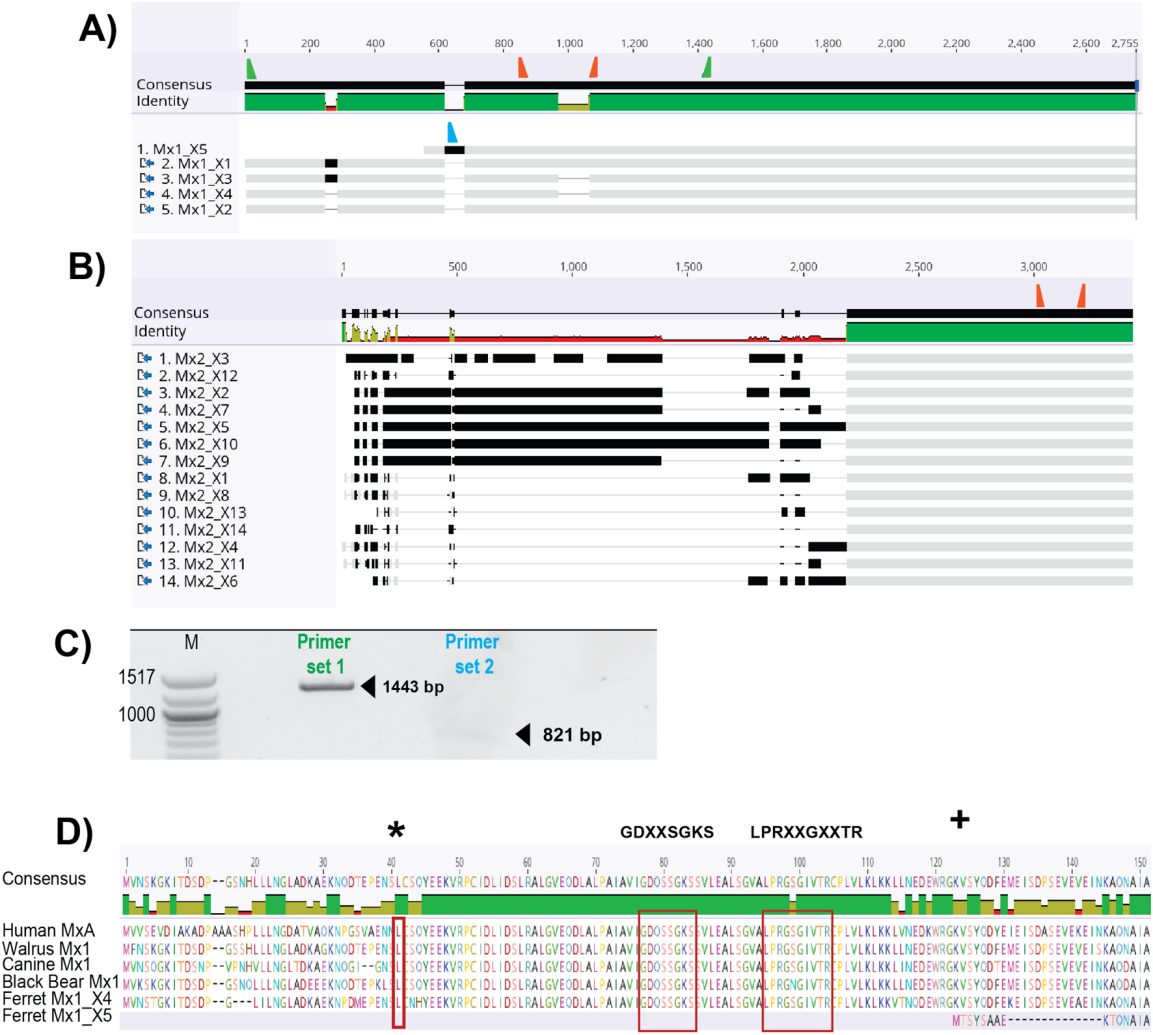
Nucleotide Alignment (MUSCLE) of predicted mRNA transcript variants of (**A**) fMx1 and (**B**) fMx2. Grey bars indicate areas of nucleotide match, black bars indicate nucleotide mismatch and grey lines show sequence deletions. In A/B, orange triangles indicate generic Mx1/2 primers used for qPCR. Note that X5 is truncated at its N-terminus relative to X1-X4. In A, green triangles indicate primers to amplify fMx1(X1-X4) (**C**, Primer set 1) and the blue triangle indicates forward primer specific for fMx1(X5) used with generic reverse Mx1 primer (green) to amplify fMx1(X5) (**C**, Primer set 2). (**C**) Agarose gel showing predicted size and actual PCR products generated from FRL cells 24 h after stimulation with ferret IFN-a using primer set 1 or 2. The full image of the gel is shown as Supplementary data 1. (**D)** Protein alignment of first 150 amino acids of different mammalian Mx proteins with translated ORFs of fMx1(X4) and (X5). Red boxes highlight leucine (L) at residue 41 (*) as well as signaling domains GDXXSGKS and LPRXXGXXTR, which are present in fMx1(X4) but not (X5). The first methionine (M) of fMx1(X5) ORF is marked with ‘ + ’.

**Figure 2:**
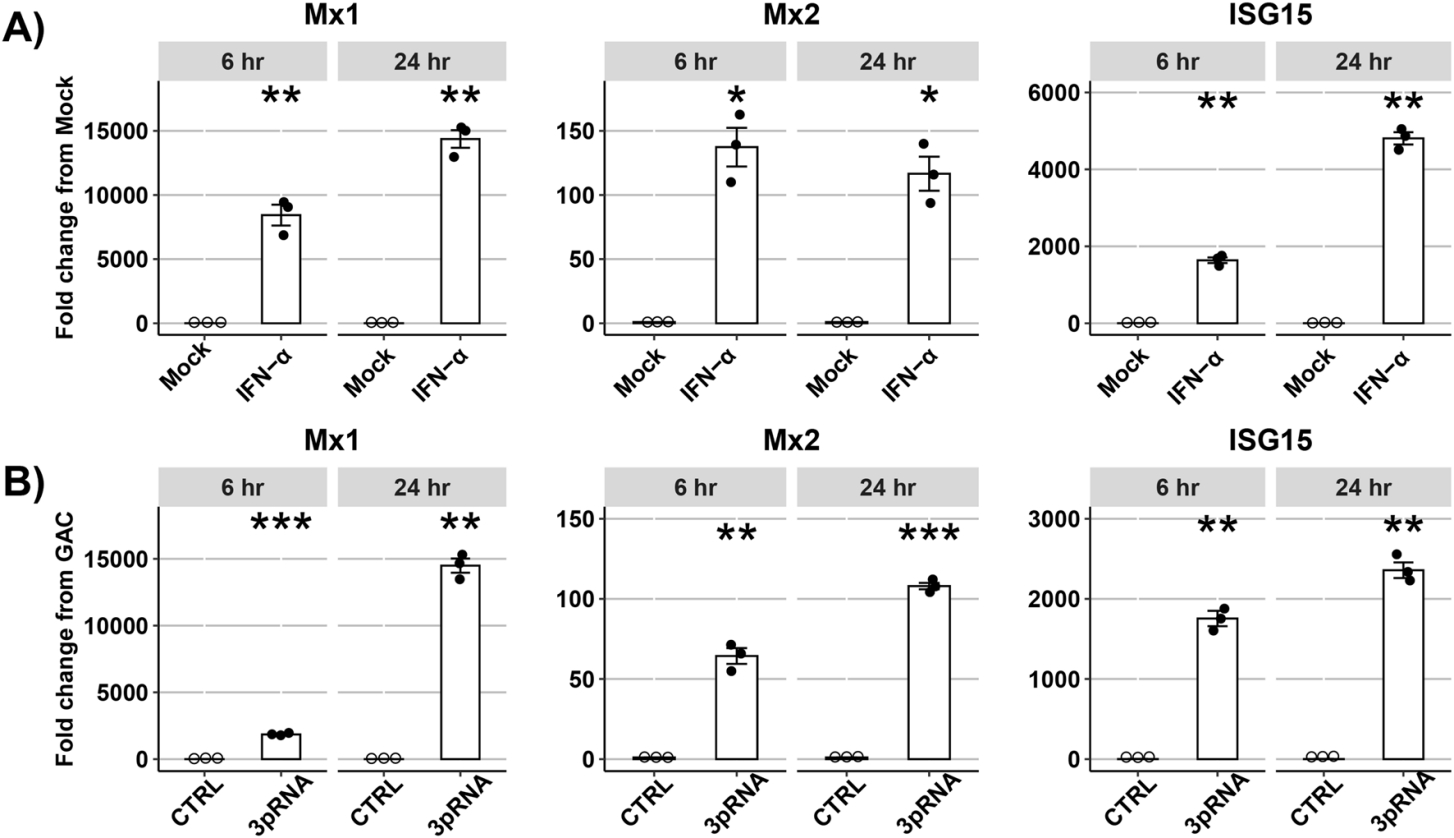
Induction of ISGs following treatment of ferret FRL cells in vitro. (**A)** FRL cells were treated with recombinant ferret IFN-a for 6 or 24 h and then total RNA was extracted and mRNA levels of ferret Mx1, Mx2, ISG15 and GAPDH (housekeeping gene) were determined by qPCR. Data show fold change relative to mock-treated cells. (**B)** FRL cells were transfected with RIG-I agonist (3pRNA) or control (ctrl) RNA for 6 or 24 h and mRNA levels of ferret ISGs and GAPDH were determined by qPCR. Data show fold change relative to ctrl. Representative data from triplicate samples in one of three independent experiments are shown. Wilcoxon Rank Sum test was used to calculate statistical differences. * *p* < 0.05, ** *p* < 0.01, *** *p* < 0.001.

**Figure 3:**
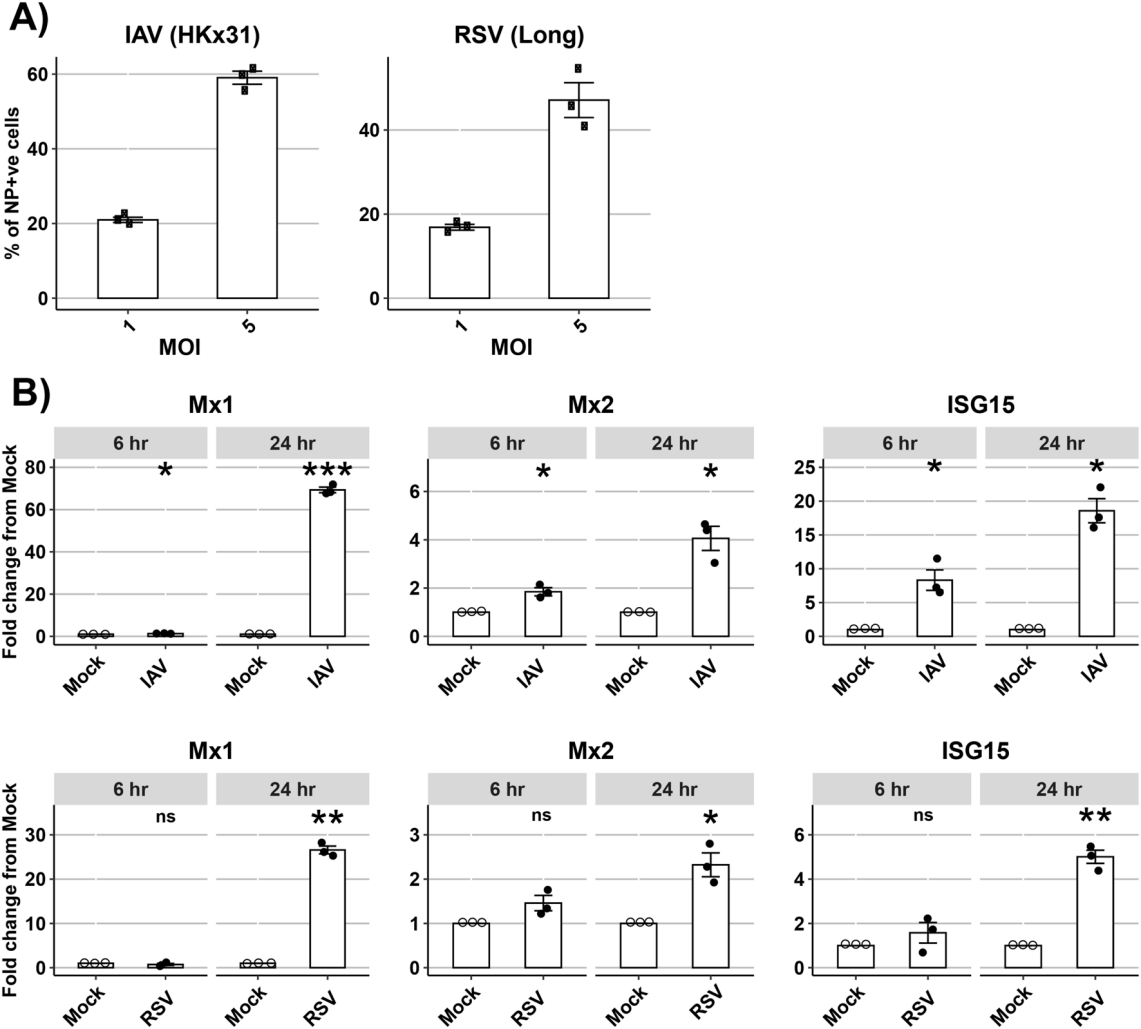
Induction of ferret ISGs following infection of FRL cells with respiratory viruses in vitro. (**A)** FRL cells were infected with IAV (HKx31) or RSV (Long) at the MOIs indicated (1 or 5) and the percentage of virus-infected cells were determined at 8 or 16 h post infection (hpi), respectively, by staining for viral NP protein and flow cytometry. Data from triplicate samples in one of two independent experiments are shown. (**B)** FRL cells were infected with either IAV (HKx31) or RSV (Long) at MOI = 5 for 6 or 24 h and then total RNA was extracted and mRNA levels of ferret Mx1, Mx2, ISG15 and GAPDH (housekeeping gene) were determined by qPCR. Upregulation of ferret ISGs was calculated relative to mock-infected cells. Representative data from one of two independent experiments with triplicate samples is shown. Wilcoxon Rank Sum test was used to calculate statistically significant upregulation of ISGs after virus infection compared to mock. * *p* < 0.05, ** *p* < 0.01, *** *p* < 0.001, ns = Not significant.

Primers were designed for qPCR to detect all variants of fMx1(X1-5) or fMx2(X1-14) (Fig. 1A,B, primers in orange). Additional primers allowed for detection of an alternative ferret ISG (fISG15), or a housekeeping gene (GAPDH). Next, the ferret lung (FRL) cell line was (i) mock-treated or treated with recombinant ferret IFN-α (Fig. 2A), or transfected with a synthetic RNA oligonucleotide to stimulate RIG-I (3pRNA) or with control (ctrl) RNA (Fig. 2B), and total RNA was isolated from cells 6 and 24 h post-treatment for qPCR. Both ferret IFN-α treatment (Fig. 2A) and 3pRNA (Fig. 2B) induced significant upregulation of fMx1 and fMx2, noting that mock or ctrl RNA treatment did not. Of note, fMx1 was induced to much higher levels than fMx2 by both IFN-α and 3pRNA (14,366 ± 687 fold (IFN-α) or 14,491 ± 533 fold (3pRNA) for fMx1 compared to 116 ± 13 fold (IFN-α) or 108 ± twofold (3pRNA) for fMx2 at 24 h post treatment). Both ferret IFN-α and 3pRNA also induced significant upregulation of fISG15 (4807 ± 160 fold (IFN-α) or 2357 ± 97 fold (3pRNA) at 24 h post-treatment).

Alignment of fMx1(X1-5) and fMx2(X1-14) transcript variants was performed (schematics shown in Fig. 1A,B, full alignment in Supplementary data 2/3). For fMx1, the X5 variant showed truncation at the N terminus (551 residues compared to the longest variant X1). Therefore, we designed primers to specifically amplify either fMx1(X1-4) (Fig. 1A, primer set 1) or fMx1 (X5 only) (Fig. 1A, primer set 2). When PCR was performed on RNA extracted from FRL cells stimulated with ferret IFN-α for 24 h, primer set 1 generated a prominent band of ~ 1400 bps whereas the band generated by primer set 2 (~ 820 bps) was substantially weaker (Fig. 1C). Moreover, translation of predicted transcripts indicated that fMx1(X5) lacked key signalling domains GDXXSGKS and LPRXXGXXTR associated with functional Mx proteins (Fig. 1D, red boxes)^6^, as well as a leucine residue at position 41 which is conserved in many Mx proteins and critical for antiviral activity against multiple RNA viruses (Fig. 1D, marked with *)^17^. While we cannot conclusively determine if fMx1_X5 is transcribed, it certainly does not appear to be the major fMx1 species produced in FRL cells in response to IFN-α.

Next, we aimed to determine the major fMx1 variant (X1-4) induced following stimulation of FRL cells with ferret IFN-α. To do this, PCR products generated using primer set 1 were cloned using the TOPO Blunt cloning kit and individual colonies were sequenced. Of these, 48/48 colonies contained fMx1(X4), and no other variants detected. Thus, fMx1(X4) which corresponds to fMx1.2 in the original annotation (NCBI XM_004762192.2), is the major fMx1 isoform expressed by FRL cells in response to IFN-α treatment. Of note, we were unable to determine the major fMx2 variant induced as we could not a design primer set that allowed for simultaneous amplification of multiple fMx2 species due to high variability in the 5’ sequence of the 14 different Mx2 variants (Fig. 1B), as well as the low levels of fMx2(X1-14) induced in FRL cells in response to ferret IFN-α (Fig. 2).

### Induction of ferret Mx1 and Mx2 in FRL cells following virus infection

Ferrets represent the ‘gold standard’ small animal model to study IAV (reviewed in^14,15^), but can also be used to study immunity and pathogenesis to human RSV infections^18^. Therefore, FRL cells were infected with IAV (HKx31 (H3N2)) or RSV (Long) at multiplicity of infection (MOI) of 1 or 5 for 1 h at 37 °C to allow for absorption and then replaced with media. The percentage of virus-infected cells was then determined by flow cytometry at either 8 or 16 hpi, respectively. Given that MOI 5 resulted in ~ 50% virus-infected cells for both IAV and RSV (Fig. 3A), FRL cells were subsequently infected with either IAV or RSV (MOI = 5) and total RNA was isolated at 6 or 24 hpi for qPCR. Compared to mock-infected cells, both IAV and RSV induced fMx1, fMx2 and ISG15 in FRL cells (Fig. 3B), noting that the fold induction was much lower for each fMx compared to 3pRNA or IFN-α treatment (Fig. 2). As for IFN-α and RIG-I agonist treatment, virus infections induced higher levels of fMx1 compared to fMx2 in FRL cells. Supernatants from non-infected Hep-2 cell supernatants, or from RSV-infected Hep2 cells depleted of virions by ultracentrifugation, showed no significant induction of ferret ISGs (ISG15, fMx1, fMx2) relative to mock (data not shown).

### *Induction of ferret Mx1 and Mx2 *in vivo* following IAV infection*

**Figure 4:**
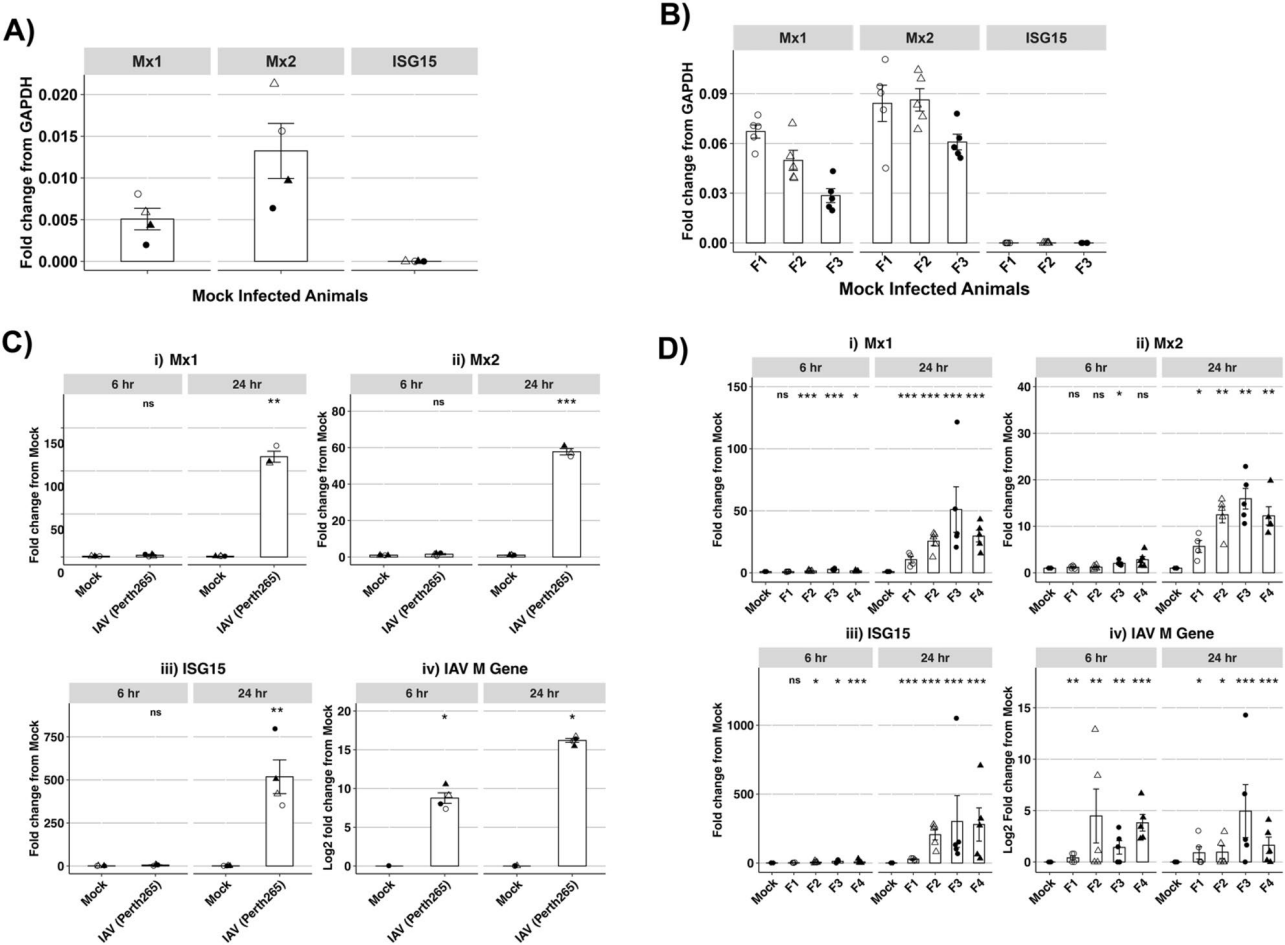
Induction of ferret ISGs in vivo following IAV infection. Ferrets (n = 3–4/group) were mock-infected or infected with with 10^7^ PFU of IAV (A/Perth/265/2009 (H1N1pdm09)) and 6 or 24 h later, lungs and nasal turbinates were removed for analysis of total RNA (by qPCR). (**A**, **B)** Baseline mRNA expression of ferret ISGs relative to housekeeping gene (GAPDH) were measured in RNA extracted from (**A)** nasal turbinates of mock-infected ferrets. Data points from individual animals are shown (n = 4) or (**B)** lung lobes from mock-infected ferrets (n = 3, designated F1, F2 and F3). Data points from 5 individual lung lobes/animal are shown. (**C**, **D)** Expression of ferret (i) Mx1, (ii) Mx2 and (iii) ISG15 (iv) IAV M gene in nasal turbinates. Data for i), ii) and iii) are shown as fold change relative to mock-treated animals (assigned a value of 1) after normalizing to housekeeping gene (GAPDH)). Data for iv) has been log transformed and expressed as fold change. (**C)** Data from individual animals are shown (n = 4/group). (**D)** Data from mock-infected animals have been pooled while data from individual IAV-infected ferrets (F1-F4, 5 lobes/animal) are shown. Statistical analysis was performed using Wilcoxon rank sum test. * *p* < 0.05, ** *p* < 0.01, *** *p* < 0.001, ns = Not significant.

Aside from FRL cells, to our knowledge few studies have utilized additional ferret cell lines for in vitro studies. To broaden the relevance of our in vitro findings in FRL cells we next examined fMx induction in the upper (nasal turbinates) and lower (lungs) airways following IAV infection. Therefore, ferrets were infected via the intranasal (i.n.) route with 10^7^ PFU of IAV, or mock-infected with an equivalent volume of PBS and, at 6 or 24 hpi, animals were euthanised and tissue from nasal turbinates and individual lung lobes were collected for RNA extraction and analysis of fMx1, fMx2 and fISG15 expression by qPCR. First, we assessed constitutive fMx expression (i.e., from mock-infected animals) by calculating the relative expression, defined as the difference in Ct values of each ISG relative to GAPDH (2^ΔCt^)^19^ (Fig. 4A,B). In both nasal turbinates (Fig. 4A) and lung lobes (Fig. 4B), fISG15 showed the lowest baseline expression relative to GAPDH, followed by intermediate expression of fMx1 and higher expression of fMx2 (Fig. 4A,B).

Next, ISG expression was assessed using the 2^ΔΔCt^ method which determines the fold change in ISG comparing test conditions (IAV infection) relative to control (mock) conditions, which is then normalized to the GAPDH housekeeping gene^19^. In addition, qPCR was performed to detect expression of IAV M gene in total RNA extracted form nasal tissues and lungs. We did not detect significant upregulation of ferret ISGs in nasal tissues from IAV-infected ferrets at 6 hpi (Fig. 4C (i/ii/iii)), despite a significant enhancement in IAV M gene in nasal tissues from all animals at this time (Fig. 4C (iv)). However, by 24 hpi we observed significant upregulation of fMx1, fMx2 and fISG15, as well as a further increase in expression of IAV M gene (Fig. 4C). Consistent with in vitro data using FRL cells, IAV infection was associated with a greater fold increase in fMx1 (100–150 fold) compared to fMx2 (50–60 fold). Relative to mock-infected animals, modest but significant upregulation of fMx1, fMx2 and fISG15 was detected in the lungs of 3/4, 1/4 and 3/4 IAV-infected animals at 6 hpi with further enhancement in each ferret ISG to significant levels in the lungs of all IAV-infected animals by 24 hpi (Fig. 4D (i/ii/iii)). Moreover, IAV infection resulted in higher levels of fMx1 induction (20–50 fold) compared to fMx2 (5–20 fold) in lungs. Detection of the IAV M gene confirmed the presence of virus in the lungs of most animals at 6 and 24 hpi (Fig. 4D(iv)).

### Homology between predicted ferret Mx proteins and Mx proteins from other species

**Figure 5:**
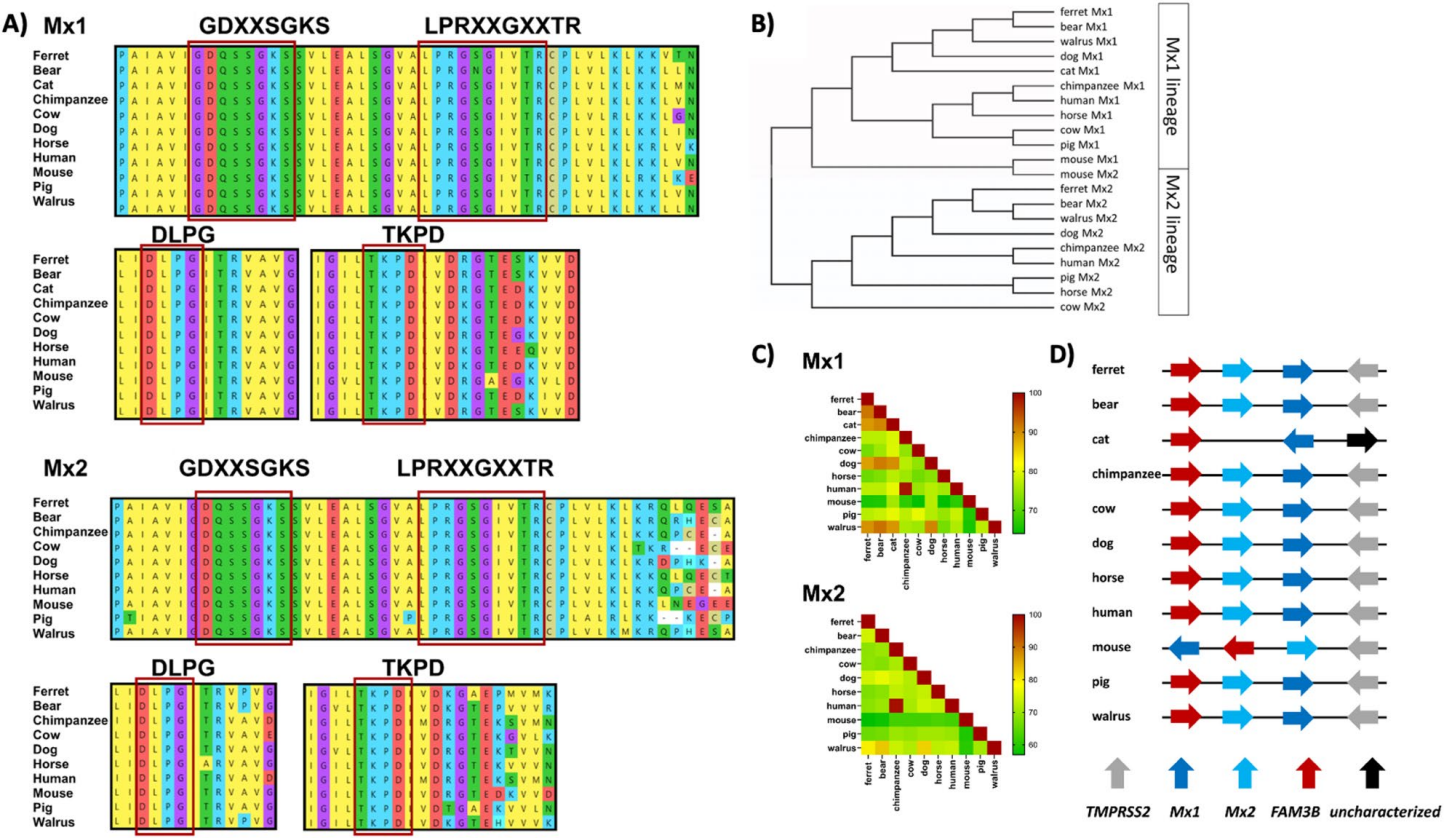
Multiple alignments of Mx1 and Mx2 proteins from different species. (**A)** Predicted amino acid sequences for species indicated were aligned by performing a MUSCLE algorithm using the Molecular Evolutionary Genetics Analysis (MEGA X) software. Conserved regions in the GTPase domain are marked. (**B**) Phylogenetic trees based on alignment from (**A**) were generated by Maximum Likelihood methods which show the relative evolutionary history of Mx proteins amongst different species. (**C**) Heatmap showing the percent homology between Mx proteins from different species, include human, mouse and ferret. (**D**) Representation of the Mx gene locus of ferrets and other mammalian species. Flanking genes are TMPRSS2 and FAM3B. Arrow direction indicates the orientation of a particular gene. For all these analyses, the following protein sequences were used: fMx1 and fMx2 (Mustela putorius furo Mx1: XP_004762249.1, Mx2: XP_012916740.1), bear (Ursus arctos horribilis Mx1: XP_026356328.1, Ursus arctos Mx2: XP_026356450), cat (Felis catus Mx1: XP_023094481.1), chimpanzee (Pan troglodytes Mx1: NP_001266765.1, Mx2 isoform X2: XP_001171751.3), cow (Bos taurus Mx1: NP_776365.1, Mx2: NP_776366.1), dog (Canis lupus familiaris Mx1: NP_001003134.1, Mx2: XP_038299486.1) horse (Equus caballus Mx1: NP_001075961.1, Mx2: XP_005606216.2), human (Homo sapiens Mx1 isoform a: NP_001138397.1, Mx2: NP_002454.1), mouse (Mus musculus Mx1: NP_034976.1, Mx2: Q9WVP9.2), pig (Sus scrofa Mx1: NP_999226.2, Mx2: A7VK00.1) and walrus (Odobenus rosmarus divergens Mx1 isoform X1: XP_004406610.1, Mx2: XP_004406645.1).

Ferret sequences used in subsequent analyses will be referred to based on the original nomenclature, namely fMx1 (NCBI XM_004762192.2, corresponding to X4) and fMx2 (NCBI XM_013061286.1). To examine the relatedness of fMx1 and fMx2 to other Mx proteins, we retrieved and aligned confirmed or predicted amino acid sequences encoded by *Mx* genes from different species. The GTPase domain of Mx proteins contains a number of conserved domains, including a tripartite GTP-binding motif (GDXXSGKS, DLPG, TKPD) and a dynamin motif (LPRXXGXXTR)^6^. Analysis of sequences for fMx1 and fMx2 confirmed that GTP-binding and dynamin signature were present in predicted ferret Mx proteins, which are associated with an intact GTPase domain and required for biological function^6^ (Fig. 5A).

Next, phylogenetic comparison of predicted protein sequences was performed based on the Maximal Likelihood method^20,21^, where Mx proteins separate into two clearly defined Mx1 and Mx2 lineages. When comparing fMx1 with Mx1 proteins from other species, we detected greatest amino acid sequence homology to Mx1 proteins from bear, walrus and dog, all of which belong to the order Carnivora (Fig. 5B). Similarly, fMx2 also clustered closely with Mx2 from bear, walrus and dog rather than with proteins from humans and mice. Note that mouse Mx1 and Mx2 form a small separate cluster which is located between the Mx1 and Mx2 lineages, with closer proximity to the Mx1 lineage as reported by others^6^. In the Carnivora, ferrets and minks both belong to the Mustelinea subfamily of the Mustelidae family. For European mink, two Mx1 (X1-2) and four Mx2 (X1-4) transcript variants have been described, noting that these are either identical to or differ by only one residue when compared to variants fMx1 or fMx2, respectively (Supplementary data 4).

We then aligned Mx protein sequences from different species by multiple pairwise alignment using NCBI protein BLAST (blastp). In these analyses, fMx1 show highest sequence homology to bear, walrus, cat and dog (Fig. 5C), confirming their close proximity as shown in phylogenetic analyses. The amino acid sequence of fMx2 was less closely related to other species using these analyses, but still showed greatest homology to Mx2 proteins from walrus, bear and dog.

In previous studies, Verhelst et al. reported the genomic organization of the *Mx* locus from different species and found that *Mx* genes are flanked by transmembrane protease serine subtype 2 (TMPRSS2) and FAM3 Metabolism Regulating Signalling Molecule B (FAM3B) genes^6^. Similar analyses confirmed that the *Mx* locus of ferrets (and most other species included into our analysis), were also flanked by TMPRSS2 and FAM3, indicating a conserved synteny across different species (Fig. 5D). Of interest, mouse *Mx* genes show a different gene orientation and arrangement, likely due to duplication of an ancestral *Mx1*-like gene^7^. Moreover, the *Mx2* locus is not present in cats.

### Cytoplasmic expression of ferret Mx1 and Mx2

Analysis of amino acid sequences using online tools cNLS mapper^22^ and NLStradamus^23^ indicated that fMx1 and fMx2 did not contain a predicted nuclear localisation sequence (NLS). NLStradamus detected a putative NLS (RKFLKERLARLGQARRRLAKF) in fMx1 however this sequence is also detected in dog Mx1 and mouse Mx2, which are both expressed in the cytoplasm^6^. Each of the programs confirmed the presence of a NLS in mouse Mx1 (REKKKFLKRRLLRLDEARQKLAKFS) and cNLS mapper also detected the NLS in human MxB (ILQEKNRYSWLLQEQSETATKRRILK), consistent with studies confirming that both of these proteins localise to the nucleus^11,24^. cNLS mapper predicts NLS-specific to the importin αβ pathway and NLStradamus can also identify additional known NLS^23^.

**Figure 6:**
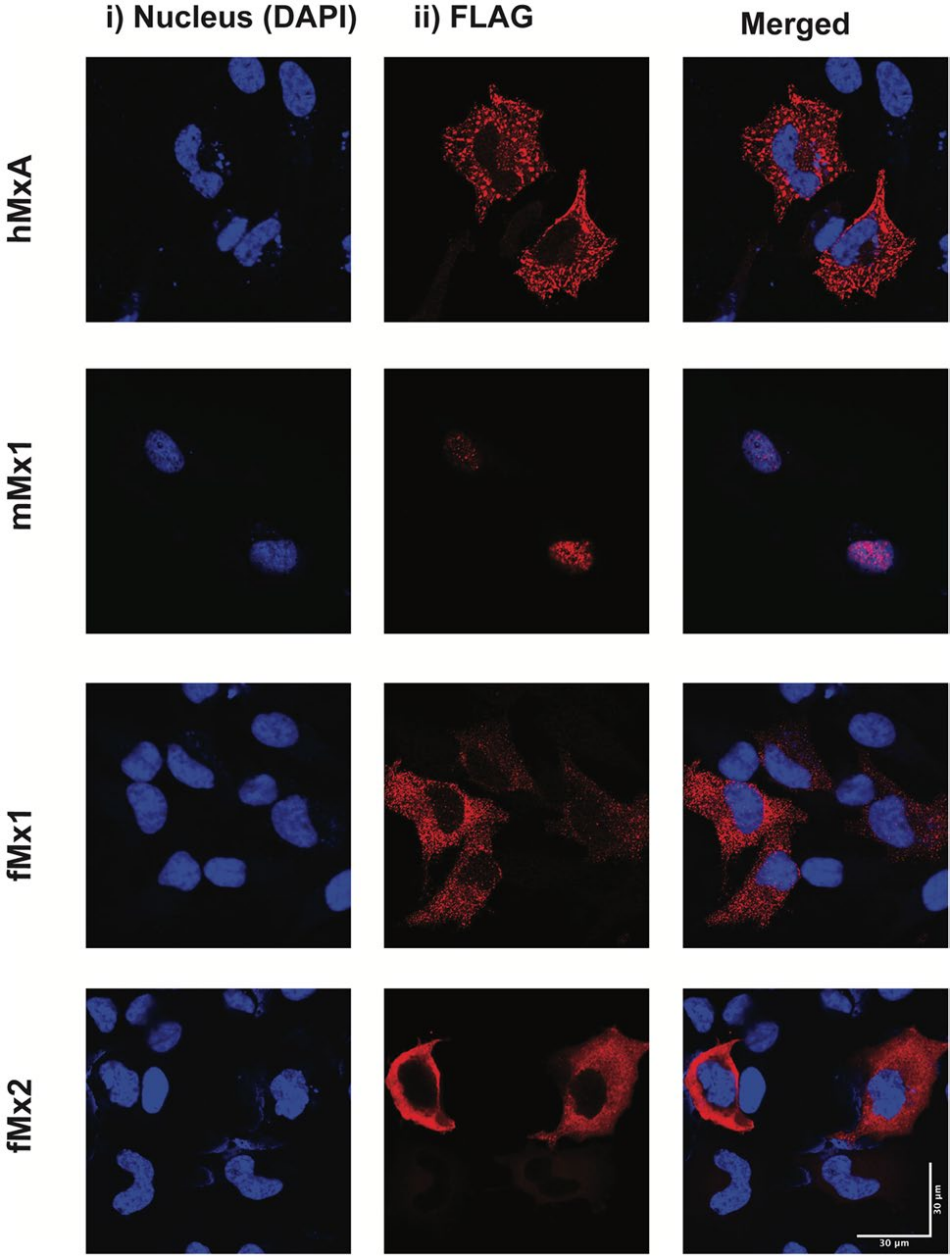
Intracellular localisation of human, mouse or ferret Mx proteins following HeLa cell transfection. Cells were transfected with pcDNA3.1 vectors expressing huMxA, mMx1, fMx1 or fMx2, each with a N-terminal FLAG tag. At 24 h post-transfection cells were fixed and stained for intracellular (**i**) DAPI and (**ii**) FLAG-tagged Mx proteins. Representative images are shown at 63 × magnification and acquired on Zeiss LSM780 confocal microscope.

Given that the power of such computational methods can be limited, we next used transfection-based approaches to determine whether fMx1 (NCBI XM_004762192.2, also known as fMx1(X4)) and fMx2 (NCBI XM_013061286.1) localised to the cytoplasm or to the nucleus. In these studies, HeLa cells were transfected with plasmids encoding different Mx proteins, each with a N-terminal FLAG tag to enable detection. As expected, transfected hMxA localised to the cytoplasm while mMx1 localised to the nucleus (Fig. 6, upper panels), consistent with their reported cellular localisation^24,25^. Like hMxA, both fMx1 and fMx2 were also expressed exclusively within the cytoplasmic compartment (Fig. 6, lower panels). Together, these studies confirm that fMx1 and fMx2 localize exclusively to the cytoplasm following expression in HeLa cells.

### Stable overexpression of ferret Mx1 can inhibit IAV infection and growth

**Figure 7:**
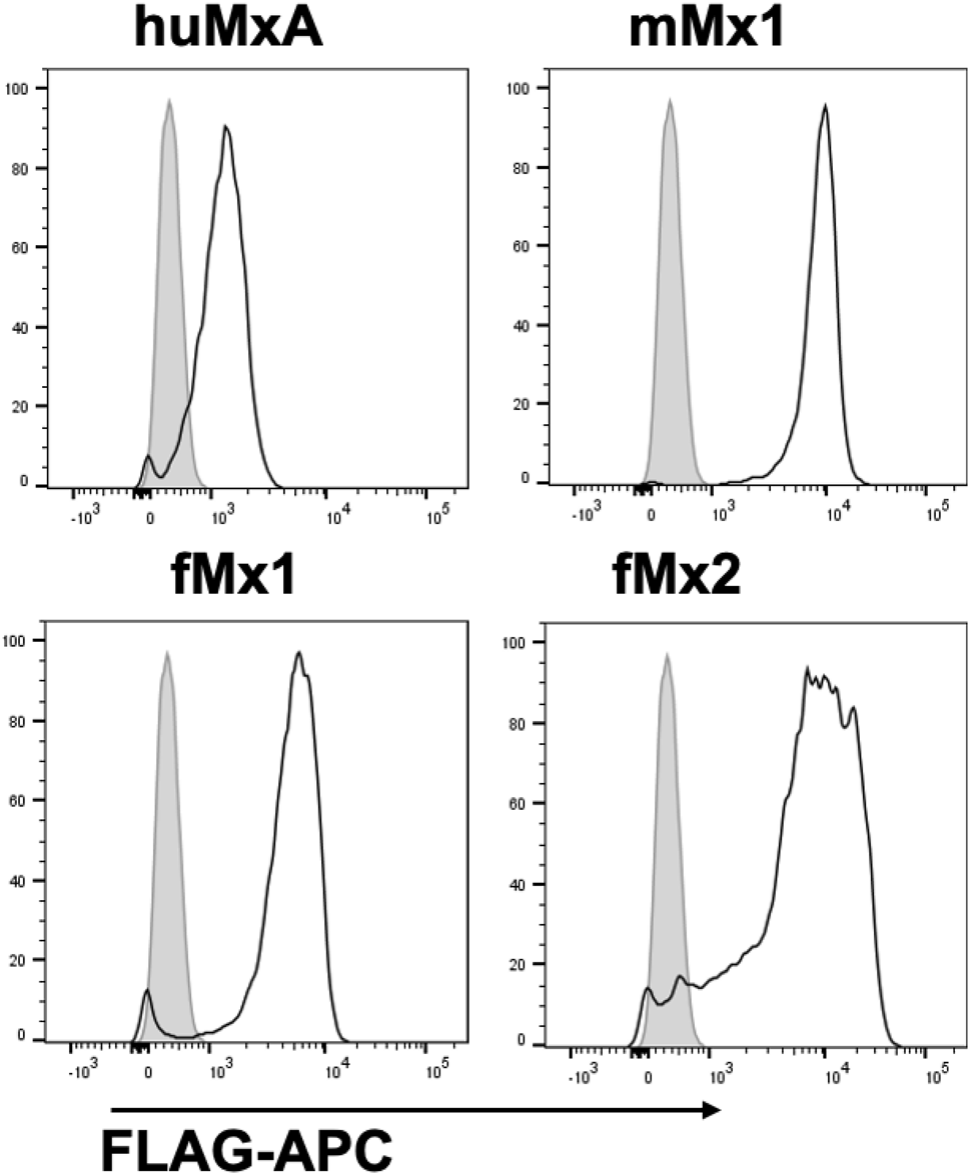
Generation of 293 T cells with stable overexpression of Mx proteins. 293 T cells were transfected with pcDNA3.1-mCherry vectors expressing huMxA, mMx1, fMx1 or fMx2, each with a N-terminal FLAG tag, or with the same vector expressing chicken ovalbumin with no FLAG tag as a control (CTRL). Stable transfectants were selected in the presence of hygromycin and enriched by sorting for mCherry^+^ cells. Cells were fixed and stained for intracellular expression of FLAG-tagged proteins and examined by flow cytometry. After gating on live mCherry + cells, FLAG expression was determined in different Mx-expressing cell lines (white histograms) relative to CTRL cells (grey histogram).

293 T cells exhibit high transfection efficiency and express exogenous proteins at high levels following transfection. Therefore, 293 T cells were transfected with pcDNA3.1 vectors encoding FLAG-tagged hMxA, mMx1, fMx1 (NCBI XM_004762192.2, also known as fMx1(X4)) or fMx2 (NCBI XM_013061286.1), or with vector encoding an irrelevant intracellular protein lacking a FLAG tag as a control (CTRL). Cell lines were then selected by culture in the presence of hygromycin and flow cytometry to enrich for mCherry^+^ cells. Flow cytometry was used to confirm high levels of intracellular FLAG staining in cell lines expressing FLAG-tagged human, mouse and ferret Mx proteins (Fig. 7).

**Figure 8:**
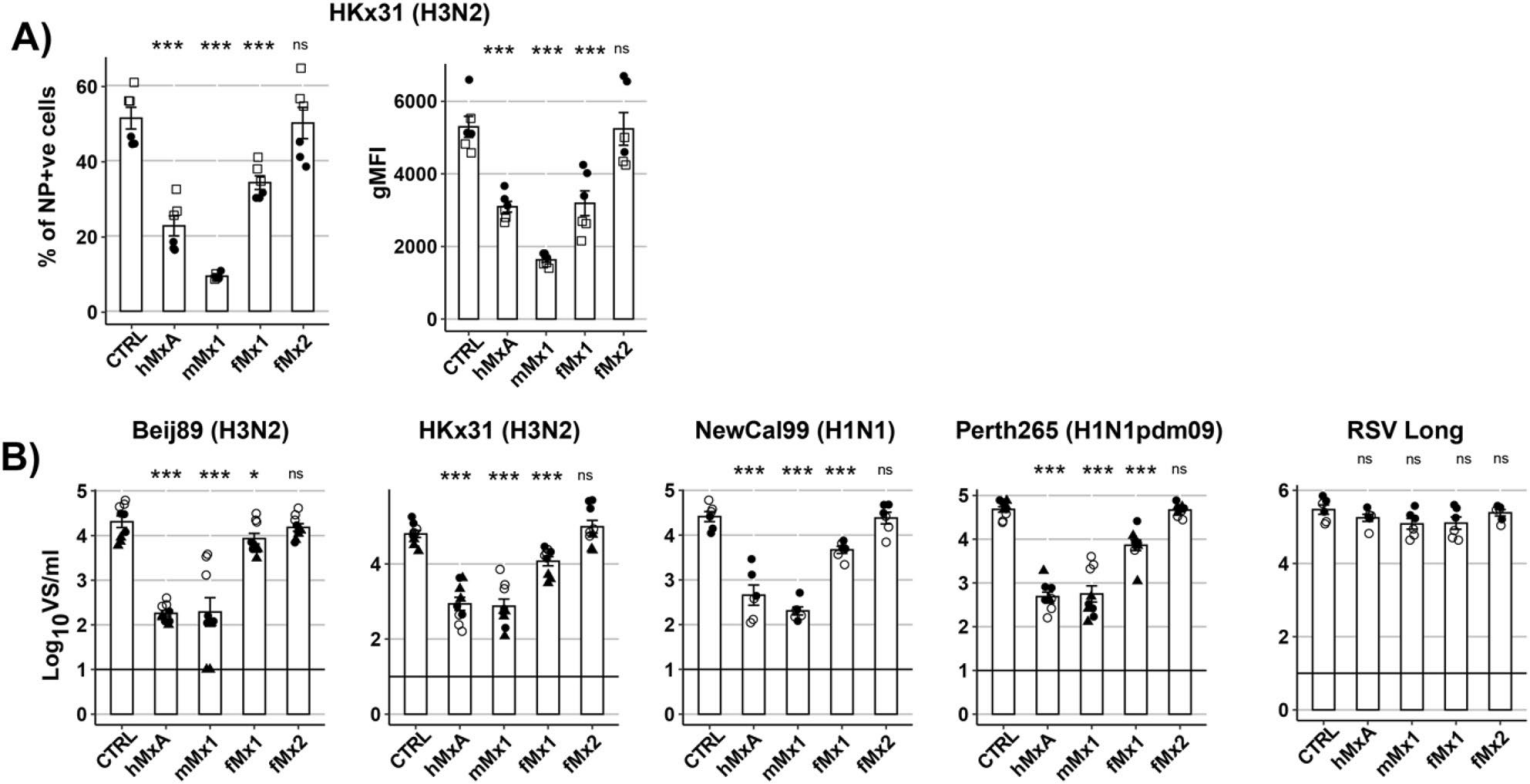
Anti-IAV activity of human, mouse and ferret Mx proteins. (**A**) CTRL 293 T cells or cells overexpressing different Mx proteins were infected with IAV (HKx31, MOI 2.5) for 8 h, then fixed and stained for intracellular expression of viral NP. Flow cytometry was performed to determine the % of NP^+^ cells and geometric mean fluorescence intensity (gMFI) of NP in the NP + gate. (**B**) Cells were infected with different IAV strains (MOI 0.1) or RSV (MOI 0.1) and titres of infectious virus in clarified supernatants were determined at 48 hpi by ViroSpot (VS) assay. For (**A**, **B**), data from two or three independent experiments, each with triplicate samples, are shown. Specific symbols correspond to triplicate samples in one experiment and symbols differ between independent experiments. Statistical analysis was performed using Student’s unpaired t-test with unequal variance to compare Mx + cell lines to the CTRL cell line. * *p* < 0.05, ** *p* < 0.01, *** *p* < 0.001, ns = Not significant.

Next, we assessed IAV infection and growth in cell lines overexpressing individual Mx proteins when compared to the CTRL cell line. Following infection with the laboratory adapted HKx31 strain (MOI = 2.5), flow cytometry confirmed reduction in the percent of IAV-infected cells in the presence of hMxA or mMx1 (Fig. 8A, upper panel), consistent with their reported antiviral activity against IAV^26^. The gating strategy used to identify NP^+^ cells is shown in Supplementary data 5. Moreover, IAV infection was also significantly reduced by overexpression of fMx1, but not fMx2, noting that inhibition by fMx1 was somewhat less potent than that observed for either hMxA or mMx1. When assessing the geometric mean fluorescence intensity (gMFI) of the viral NP in NP^+^ cells, overexpression of either hMxA, mMx1 or fMx1 also significantly reduced the levels of viral NP expression in the remaining infected cells detected whereas fMx2 did not (Fig. 8A).

To determine the impact of Mx overexpression on IAV growth, cells were infected with different IAV strains at low MOI (0.1 PFU/cell) in the presence of exogenous trypsin to promote multicycle virus replication (Fig. 8B). In these studies we utilised HKx31, as well as representative human IAV strains of the A/H3N2, A/H1N1 and A/H1N1pdm09 subtypes as human seasonal strains are routinely used to infect ferrets in published studies. Relative to virus titres recovered from CTRL cells at 48 hpi, all IAV strains tested were potently inhibited by overexpression of hMxA or mMx1 (> 90% reduction in virus titre). Overexpression of fMx1 resulted in a more modest, but significant, reduction in virus growth for 3/3 of the human IAV, while overexpression of fMx2 did not result in inhibition of any IAV strain tested. Consistent with previous reports^27^, growth of RSV was not inhibited by overexpression of any of the different Mx proteins tested.

## Discussion

Since ferrets were first discovered to be sensitive to influenza infection in the 1930s they have been used to study various aspects of infection with human and avian IAV. In addition to influenza, viral diseases associated with distemper virus, coronaviruses, parvoviruses and rabies virus, amongst others, also occur naturally in ferrets (reviewed in^28^). For IAV, ferrets have been widely used to study pathogenesis and immunity, including the impact of vaccination and/or antiviral treatments. However, innate responses to IAV and other viruses have not been well characterised in ferrets, largely due to a lack of immunological reagents which limit insights to transcriptional, rather than to functional, innate responses to infection.

Mx proteins are important components of innate cell-intrinsic antiviral immunity and Mx proteins from different species have been shown to mediate antiviral activity against a range of viruses. In some cases, Mx proteins act against a similar spectrum of viruses, however other studies suggest considerable inter-species variation in the viruses inhibited by Mx proteins. Currently, very little is known regarding the induction and anti-IAV activity mediated by any ferret ISG proteins. Mx proteins from a number of species, including humans, mice, bats, pigs and chickens, have been reported to restrict IAV infection in vitro (reviewed in^6^), but no such studies exist regarding ferret Mx proteins. Herein, we report induction of ferret Mx1/2 in vitro and in vivo, as well as their intracellular localisation and antiviral activity against IAV.

Initially, we designed qPCR primers to detect all predicted transcript variants of fMx1(X1-5) and fMx2 (X1-14) and used these to confirm induction of fMx1/fMx2 in FRL cells. While both fMx1 and fMx2 were induced in response to ferret IFN-α, RIG-I stimulation or IAV infection, fMx1 was induced to much higher levels and this facilitated the identification of fMx1(X4) as the major variant produced in response to IFN-α. This finding addresses a gap in our current understanding regarding which of predicted fMx1 transcript variants in the NCBI database is truly expressed under biological conditions. Enhanced induction of fMx1 was also observed following IAV infection of FRL cells in vitro and in nasal turbinates and lungs from IAV-infected ferrets. Currently, the major fMx2 species produced by ferret cells in vitro or in vivo is unknown. Reduced potency of induction, combined with high sequence variability in the N-terminal sequence of fMx2(X1-14), hindered our efforts to identify the major fMx2 transcript variant produced.

Our studies then focussed on further characterisation of fMx1. For comparison, we did include the original fMx2 (NCBI XM_013061286.1) in subsequent overexpression studies assessing the subcellular localisation and antiviral activity of ferret Mx proteins, however further studies are required to confirm which fMx2 variant represents the major species produced in response to IFN-α and/or different viral infections. Of note, polymorphisms have been reported in Mx from a number of species at both the genetic and the transcript level, including in pigs^29^, cattle^30,31^ and chickens^32^, amongst others. The chicken genome has only one Mx and studies with chicken Mx have been controversial, with 25 nucleotide substitutions detected, 14 of which were predicted to result in amino acid changes^32^ and inconsistent results from studies investigating whether the single nucleotide polymorphism encoding residue 631 of chicken Mx does^32,33^ or do not^34^ determine anti-IAV activity.

Previous immunohistochemistry studies using a mAb to hMxA detected a cross reactive protein with cytoplasmic expression in lung sections from H5N1-infected ferrets^35^. Herein, we used transfection-based approaches to confirm that both fMx1 and fMx2 proteins localised to the cytoplasmic compartment. Moreover, overexpression of fMx1 was associated with inhibition of IAV infection (as measured by synthesis of viral NP in infected cells), as well as growth of multiple human (> 50% reduction in viral titres) (Fig. 8). While significant, inhibition of IAV by fMx1 was relatively modest when compared to hMxA or mMx1, despite similar levels of protein expression (Fig. 7). Moreover, we did not observe significant inhibition of any of the IAV strains tested in stable cell lines overexpressing fMx2. These results are consistent with the involvement of fMx1 in the RIG-I-mediated antiviral effects we have previously reported in IAV-infected ferrets^3^.

To date, relatively little is known regarding the anti-IAV activity of Mx1/2 proteins expressed by species in the suborder Caniformia (‘dog like’ carnivorans). In addition to dogs, bears and raccoons, mustelids form the largest family and include ferrets, weasels, badgers and mink. Consistent with this, predicted protein sequences for fMx1 and fMx2 were more closely related to other species of the suborder Caniformia than to human or mouse Mx proteins (Fig. 5). Within Caniformia, it appears that only the antiviral activity of canine Mx proteins has been investigated in a limited number of studies to date. For example, overexpression of canine Mx2, but not Mx1, significantly reduced the percentage of VSV-infected 3T3 cells^36^. Recently, Bayrou et al. demonstrated that 293 T cells overexpressing canine Mx1 showed reduced levels of infection by Schmallenberg virus, a bunyavirus of veterinary significance, although inhibition was modest when compared to that mediated by equine, porcine or bovine Mx1^37^. In terms of IAV, current literature suggests that neither canine Mx1, nor Mx2, inhibit IAV polymerase activity^38^, nor does canine Mx1 inhibit IAV infection^39^. Thus, while it is clear that viruses of equine (A/H3N8) and avian (A/H3N2) origin can circulate efficiently in dog populations worldwide (reviewed in^40^), canine Mx proteins do not appear to represent a major component of antiviral innate immunity to IAV.

For hMxA, the generalised function of the loop L4 domain (residues 533 to 572) and, in particular, a phenylalanine at residue 561 is critical for its antiviral activity against orthomyxoviruses^41^. Indeed, the presence of an aromatic residue at residue 561 or 562 has been proposed to correlate with potent anti-IAV activity of Mx1 proteins from species such as horses, pigs and cows^39^. However, Mx1 proteins from Caniformia species including walrus, bear, dog (which does not inhibit IAV) and ferrets express a methionine at residue 561. Alignment of the L4 loop of fMx1 and canine Mx1 shows differences in 13 residues, including a phenylalanine at position 567 of fMx1 which is absent in canine Mx1. While distant from residue 561, whether this still contributes to the anti-IAV activity of fMx1 remains to be determined. Our findings with fMx1 suggest that there is more complexity beyond the presence or absence of an aromatic residue at position 561 or 562 in determining the ability of different Mx1 proteins to restrict IAV replication. Swapping L4 loops between fMx1, canine Mx1 and hMxA, or mutagenesis to add (561) or delete (567) particular aromatic residues in fMx1, will provide further insight regarding modulating the potency of anti-IAV activity in Mx1 proteins from different species.

Studies reported herein provide the first key insights regarding the regulation, localisation and antiviral activity of fMx1, noting that previous studies have performed comprehensive transcriptional analyses of ISGs, including fMx1 and fMx2, in the blood and lungs of IAV-infected ferrets^42^. Our finding that fMx1 can inhibit IAV infection and replication, despite its similarity to canine Mx1 (which does not inhibit IAV), provides avenues for further research to determine the influence of specific Mx1 regions and residues which modulate the potency of anti-IAV activity. While short read sequencing has been used to determine predicted fMx transcript variants currently reported in NCBI, future studies using long read sequencing to determine full length ferret mRNA transcripts will allow for identification of the predominant variants induced in biological settings. Determining the major transcript variant of fMx2, as well as the spectrum of antiviral activity of fMx1 and fMx2 against additional viruses, including those that naturally infect ferrets, will also provide important insights regarding the relative contributions of each ferret Mx to innate antiviral immunity in ferrets. Given the recent surge of IAV infections in farmed mink (reviewed in^43^), comparing the anti-IAV activities of the closely related ferret and mink Mx1/2 is also of interest, noting that European mink Mx1(X2) (NCBI XP_059020753.1) is identical to fMx1.2 (also known as X4) which we have shown to be the predominant transcript produced by FRL cells in response to IFNα.

Recently, Horman et al. reported induction of ferret IFITM1, 2 and 3 in vitro in response to treatment of cells with ferret IFN-α or infection with IAV^44^, although these studies did not investigate induction in vivo, cellular localisation or the antiviral activity of the different ferret IFITMs. Further studies characterising similarities and differences between restriction factors expressed by humans and ferrets are of particular importance given the widespread use of ferrets as animal models to study IAV and other human respiratory virus infections, and of new prophylactic and therapeutic approaches that are based on activation of innate immune receptors such as RIG-I^1–4^.

## Methods

### Cells and viruses

Human embryonic kidney (HEK)293 T cells (American Type Culture Collection (ATCC), CRL-3216), cervical carcinoma HeLa cells (CCL-2), HeLa-derived human epithelial type 2 (HEp-2) cells (CCL-23), and FeRret Lung (FRL) cells were maintained and passaged in Dulbecco's Modified Eagle Medium (DMEM) (Gibco) supplemented with 10% (v/v) FBS, 2 mM L-glutamine (Gibco) and 1 mM sodium pyruvate (Gibco). FRL cells are an adenovirus 5 immortalized cell line kindly provided by Dr. Tuck-Weng Kok, University of Adelaide, Australia. Madin-Darby Canine kidney (MDCK) cells (ATCC CCL-34) were maintained and passaged in RPMI 1640 medium supplemented with 10% (v/v) fetal bovine serum (FBS, Gibco), 2 mM L-glutamine and 1 mM sodium pyruvate.

Seasonal IAV strains were used in this study were A/Beijing/353/89 (Beij89, H3N2), A/New Caledonia/20/99 (NewCal99, H1N1), A/Perth/265/2009 (Perth09, H1N1pdm09) and A/HKx31 (HKx31, H3N2), a high-yielding reassortant of A/Aichi/2/68/60 (H3N2) with PR8 bearing the H3N2 surface glycoproteins. All influenza viruses were obtained from the WHO Collaborating Centre for Reference and Research on Influenza (WHO CCRRI), Melbourne, Australia. Viruses were obtained from the WHO Collaborating Centre for Reference and Research on Influenza, Melbourne, Australia and were propagated in the allantoic cavity of 10-day embryonated chicken eggs (with approval from the University of Melbourne Biochemistry & Molecular Biology, Dental Science, Medicine, Microbiology & Immunology, and Surgery Animal Ethics Committee, project #10448), following standard procedures^45^ and titres of infectious virus were determined on MDCK cells by standard plaque assay and expressed as plaque-forming units (PFU) per mL^46^. RSV strains used for this study was RSV Long (VR-26, purchased from ATCC). RSV stocks were generated by propagation in HEp-2 cells and quantified on HEp-2 cells by Virospot (VS) assay as described^18^.

### *Treatment or infection of FRL cells *in vitro

FRL cells were seeded in 24-well plates 24 h prior to treatment with 50 ng/ml recombinant ferret IFN-α (a kind gift from Tim Adams, CSIRO Manufacturing, Parkville, Australia), transfection with RIG-I agonist or infection with human respiratory viruses. Synthetic 3pRNA, consisting of a 5’-triphosphorylated, double-stranded hairpin RNA, was chemically synthesized by solid-phase synthesis using product-specific labelling, as described^47^. Control (ctrl) RNA was also generated^47^. For stimulation of RIG-I, Lipofectamine 2000 (Invitrogen) was used to transfect FRL cells with 3pRNA (100 ng/mL) or ctrl RNA (100 ng/mL) in Opti-MEM (Gibco-BRL) according to manufacturer’s instructions. At 6 or 24 h post treatment, total RNA was extracted from cells using the RNeasy Plus Mini Kit (Qiagen), and treated with amplification grade DNase I (Sigma Aldrich). Generation of cDNA and qPCR for ferret ISGs were performed as described below.

To determine susceptibility of FRL cells to IAV or RSV infection, cells seeded in 24-well plates were cultured overnight and then infected with either IAV (HKx31) or RSV (Long) for 1 h at 37 °C, washed and then cultured for an additional 7 h (for IAV) or 15 h (for RSV). After incubation, cells were detached, washed, stained with the fixable viability dye eFluor780 (eBioscience), and then fixed with 4% (vol/vol) paraformaldehyde (PFA) in water, permeabilised with 0.5% (vol/vol) Triton-X 100(Sigma-Aldrich) in phosphate-buffered saline (PBS) and stained in PBS containing 0.25% (vol/vol) Triton-X 100, 1% FCS and 1 mM EDTA. For IAV, cells were stained with a fluorescein isothiocyanate (FITC)-conjugated monoclonal antibody (mAb) specific for IAV NP (clone D67J, Thermofisher Scientific). For RSV, cells were stained with a mAb specific for RSV NP (clone B023, Invitrogen) followed by a Alexa Flour™ 488 chicken anti-mouse antibody (clone A21-200, Invitrogen). Data acquisitions were performed on BD LSR Fortessa (BD Bioscience). In some experiments, FRL cells were infected with IAV or RSV for 6 or 24 h, before total RNA was extracted and treated with amplification grade DNase I. Generation of cDNA and qPCR for ferret ISGs were performed as described below.

### Ethics statement

Ferret experiments were conducted with approval from the University of Melbourne Biochemistry & Molecular Biology, Dental Science, Medicine, Microbiology & Immunology, and Surgery Animal Ethics Committee (AEC# 20033), in accordance with the NHMRC Australian code of practice for the care and use of animals for scientific purposes.

### IAV infection of ferrets

Adult outbred ferrets (600–1500 g) were housed in the Bioresources Facility at the Peter Doherty Institute for Infection and Immunity, Melbourne, Australia. Prior to the commencement of experiments, hemagglutination inhibition assays were used to confirm all animals to be seronegative against IAV strain A/Perth/265/2009 ((H1N1)pdm09). For IAV infection, 12 ferrets were anaesthetised (25 mg/kg ketamine and 5 mg/kg ilium Xylazilin a 1:1 (vol/vol) mixture) and 8 were inoculated by dropwise intranasal delivery of 500uL of PBS containing 10^7^ PFU of Perth09 and 4 were mock-infected with an equivalent volume of PBS. At either 6 or 24 h post infection, ferrets were euthanized for collection of nasal tissues and 5 lung lobes from each animal. For euthanasia, ferrets were anesthetised using a mixture of ketamine and xylazine following pentobarbitone sodium (Lethabarb Troy Laboratories) injection. Sections of individual lung lobes and nasal tissues were stored in 5 mL RNALater overnight at 4 °C and tissues were then frozen at − 80 °C. Total RNA was extracted from ferret respiratory tissue samples using the RNeasy Mini Plus kit (Qiagen) according to the manufacturer's instructions. Briefly, 5 ml or 3 ml RLT buffer containing 143 mM β-mercaptoethanol was added directly to lung lobe sections and nasal turbinates, respectively, in gentleMACS M tubes (Miltenyi Biotec). Samples were homogenized using the gentleMACS dissociator (Miltenyi Biotec) and lysates were clarified twice by centrifugation at 3,000 × *g* for 10 min. RNA was extracted using the animal tissue protocol and eluted in 50 μl. RNA concentration and purity was assessed by spectrophotometry (*A*_260_/*A*_280_). Generation of cDNA and qPCR for ferret mRNA were performed as described below.

### Detection of ferret ISGs by qPCR

After standardization, RNA was converted to cDNA using the SensiFAST cDNA Synthesis Kit (Bioline). SYBR green-based qPCR was used to determine expression of ferret Mx1, Mx2 and ISG15 relative to housekeeping gene GAPDH (glyceraldehyde 3-phosphate dehydrogenase) using the SensiFAST SYBR. Data acquisition was performed using the QuantStudio 7 Flex Real-Time PCR System (Applied Biosystems). The primers used were as follows. Generic fMx1: Fwd 5′-ACATCCTCAGGCAGGAGACA-3′, Rev 5′-CAGGTCAGGCTTTGTCAAGA-3′; Generic fMx2: Fwd 5′- AAGTGGCTCAGAACCTCACG-3′, Rev 5′-GTCAGTCTTTCCGCCAGACA-3′; fGAPDH: Fwd 5′-AACATCATCCCTGCTTCCACTGGT-3′, Rev 5′-TGTTGAAGTCGCAGGAGACAACCT-3′; fISG15: Fwd 5′-AGCAGCAGATAGCCCTGAAA-3′, Rev 5′-CAGTTCTTCACCACCAGCAG-3′.

To detect particular fMx1 transcripts upregulated in response to ferret IFN-α, FRL cells in 6-well plates were treated with recombinant ferret IFN-α (50 ng/ml) for 24 h and then total RNA was extracted and converted to cDNA. PCR was then performed using primers fMx1 set 1 (X1-4), Fwd 5′-GCATAAAAGCGGAGGGGCTG-3′, Rev 5′-GTCTTCGGGCACATCTGAGC-3′ or fMx1 Set 2 (X5), Fwd 5′-GGAAGTGGGTTCATAGCCGC-3′, Rev 5′-GTCTTCGGGCACATCTGAGC-3′ and the iProofTM High-Fidelity DNA polymerase kit (Biorad) and PCR products were run on a 1% agarose gel and visualized using Sybr Safe DNA gel stain (Invitrogen). PCR products generated using primer set 1 fMx(X1-4) were then cloned into pCR™Blunt II-TOPO™ vector using the Zero Blunt™ TOPO™ PCR Cloning Kit (Invitrogen), and transformed into DH5α™-T1R (Invitrogen, cat K2895-20) chemically competent cells as per manufacturer’s instructions. After transformation, 50 colonies were picked and sequenced using M13 forward and reverse primers provided.

### Bioinformatic analysis of ferret Mx proteins

Sequences of Mx1 and Mx2 proteins from different species were obtained from NCBI databases. MEGA X software was used to perform MUSCLE algorithm and Maximal Likelihood method. NCBI protein BLAST (blastp) was used to determine amino acid sequence proximities.

### Generation of 293 T cell lines with stable constitutive expression of Mx proteins

Genes encoding mMx1, huMxA, fMx1.2 (NCBI XM_004762192.2, also known as fMx1(X4)) or fMx2 (NCBI XM_013061286.1), each with a N-terminal FLAG tag , or cytoplasmic ovalbumin lacking a FLAG tag as a control (CTRL), were cloned into pcDNA3.1 expression plasmids (Invitrogen) modified to express the IRES mCherry element as described^48^. After cloning, inserts were confirmed by sequencing and 1 μg of each plasmid were transfected into 293 T cells using Lipofectamine 2000 (Life Technologies, CA, USA). Cells were cultured at 37 °C for 2 days, before media was supplemented with 100 μg/mL hygromycin B (InvivoGen). After culture for > 10 days, mCherry-positive cells were sorted twice to enrich for mCherry-positive cells before use in subsequent experiments.

Flow cytometry was used to detect intracellular expression of FLAG-tagged proteins in 293 T cells. Cells were stained with with fixable viability dye eFluor 450 (eBioscience) and then fixed, permeabilised and stained with allophycocyanin (APC)-conjugated anti-FLAG MAb (clone L5; Biolegend) prior to analysis by flow cytometry.

### Confocal microscopy to detect subcellular localisation of ferret Mx proteins

Localisation of FLAG-tagged mMx1, huMxA, fMx1 (NCBI XM_004762192.2, also known as fMx1(X4)) and fMx2 (NCBI XM_013061286.1) were visualized in Hela cells by confocal microscopy. Cells were seeded into 12 mm coverslips circles (Deckglaser, Menzel-Glaser), cultured overnight and then transfected with 2ug of pcDNA3.1-mCherry vectors expressing different FLAG-tagged Mx proteins using Lipofectamine 2000 (Invitrogen) and incubated for 24 h. Cells were then fixed in phosphate-buffered saline (PBS) containing 4% (vol/vol) paraformaldehyde (PFA) before permeabilization in PBS containing 5% (wt/vol) bovine serum albumin, 5% (vol/vol) FCS, and 0.1% (vol/vol) Triton X-100. FLAG expression was detected by using anti-FLAG mAb conjugated to Alexa Fluor 647 (L5; BioLegend). Prior to visualization, cells were counterstained with Vectashield antifade mounting medium containing 4′,6-diamidino-2-phenylindole (DAPI) (Vector, Burlingame, CA, USA). Images were acquired with a Zeiss LSM780 microscope and were processed using Fiji ImageJ software.

### Virus infection and growth in cells overexpressing Mx proteins

Cells seeded in 24-well tissue culture plates were cultured overnight, washed and then inoculated with IAV at the indicated multiplicity of infection (MOI, in PFU/cell). After incubation for 1 h at 37 °C, cells were washed to remove residual inoculum and incubated in serum-free media. In some experiments, media was supplemented with 0.5 μg/mL TPCK trypsin (Worthington Biochemical) to promote multicycle replication. To evaluate the early stages of virus replication, the percentage of cells expressing newly-synthesised IAV nucleoprotein (NP) was measured by flow cytometry at 8 hpi^48^. Briefly, virus-infected cells were stained with fixable viability dye eFlour 780 (eBioscience), and then fixed, permeabilized and stained with anti-IAV NP conjugated to FITC (Clone 431, Abcam) before analysis by flow cytometry. To evaluate IAV replication, cell supernatants were collected at indicated times, clarified by centrifugation and titres of infectious virus in cell-free supernatants determined by ViroSpot (VS) immunostain assay as described^49^.

### Statistical analysis

Graphs and statistical analysis (as indicated in the figure legends) were performed using R software version 4.0.3 . Flow cytometry data was analysed using FlowJo version 10 (Becton, Dickinson and Company).

### Supplementary Information

Supplementary Information 1.

Supplementary Information 2.

Supplementary Information 3.

Supplementary Information 4.

Supplementary Information 5.

## Data Availability

Data is provided within the manuscript or supplementary information files. Any additional data that support the findings of this study are available from the author, P.C.R., upon reasonable request.
