# Supplementary figures and images for: Induction and antiviral activity of ferret myxovirus resistance (Mx) protein 1 against influenza A viruses
Source: Sci Rep. 2024 Jun 12;14:13524. doi: 10.1038/s41598-024-63314-2 (PMC11169552; doi:10.1038/s41598-024-63314-2)

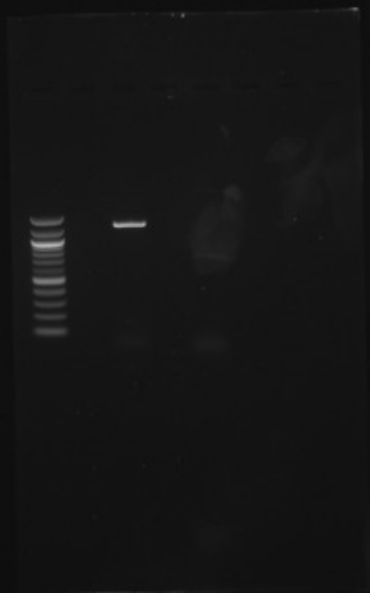

Supplement: Supplementary file 1 — Supplementary Information 1. [file 41598_2024_63314_MOESM1_ESM.pdf]
