# Supplementary material for: Induction and antiviral activity of ferret myxovirus resistance (Mx) protein 1 against influenza A viruses
Source: Sci Rep. 2024 Jun 12;14:13524. doi: 10.1038/s41598-024-63314-2 (PMC11169552; doi:10.1038/s41598-024-63314-2)
Supplement: Supplementary file 4 — Supplementary Information 4. [file 41598_2024_63314_MOESM4_ESM.pdf]

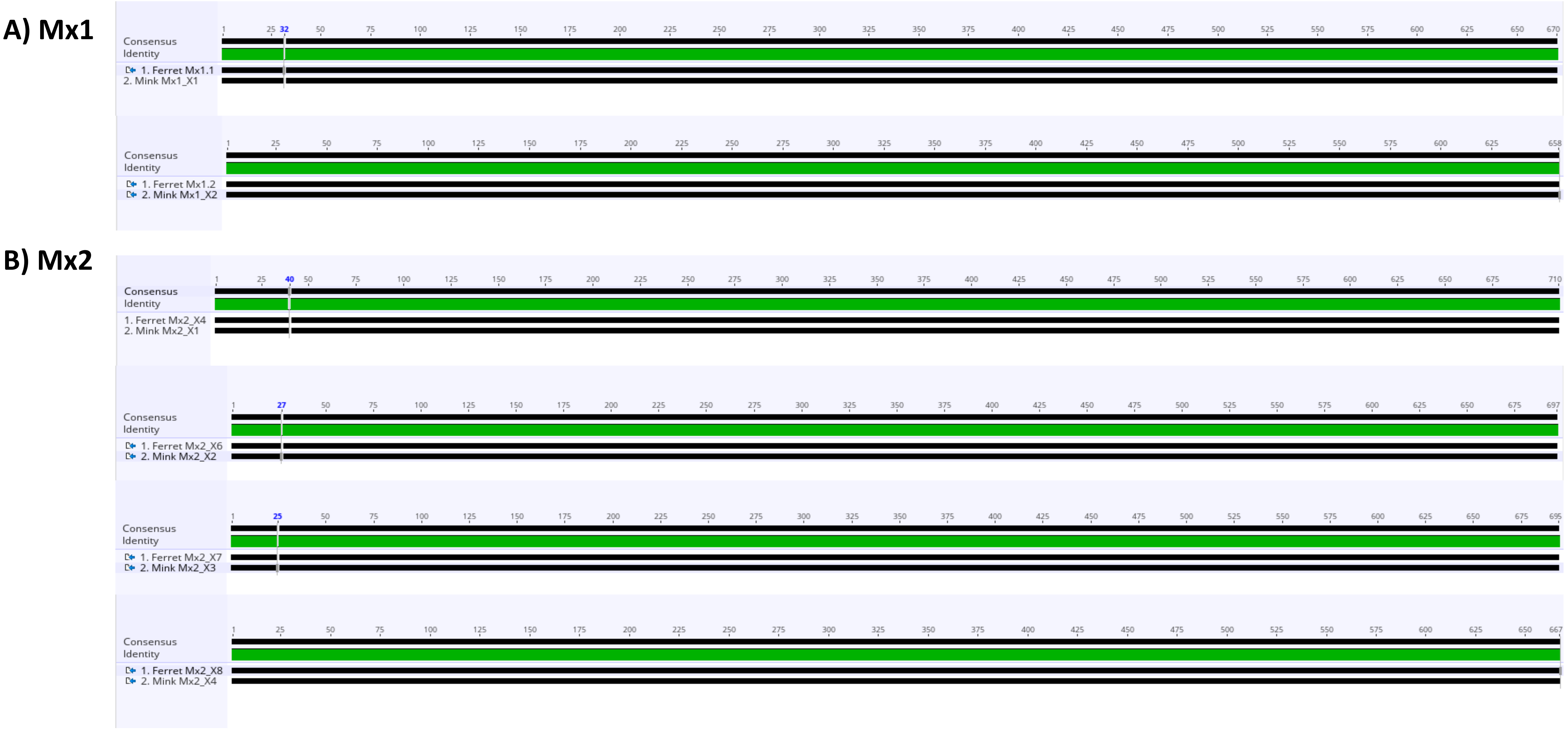

Supplementary figure

A) Protein alignment of ferret Mx1.1 with European mink (Mustela lutreola) Mx1_X1 (XP_059020752.1) and ferret Mx1.2 with mink Mx1_X2 (XP_059020753.1). **B)** Protein alignment of ferret Mx2_X4 with mink Mx2_X1 (XP_059020746.1), ferret Mx2_X6 with mink Mx2_X2 (XP_059020747.1), ferret Mx2_X7 with mink Mx2_X3 (XP_059020748.1) and ferret Mx2_X8 with mink Mx2_X4 (XP_059020749.1). Blue numbers indicate position of amino acid mismatch.
