# Supplementary material for: Induction and antiviral activity of ferret myxovirus resistance (Mx) protein 1 against influenza A viruses
Source: Sci Rep. 2024 Jun 12;14:13524. doi: 10.1038/s41598-024-63314-2 (PMC11169552; doi:10.1038/s41598-024-63314-2)
Supplement: Supplementary file 5 — Supplementary Information 5. [file 41598_2024_63314_MOESM5_ESM.pdf]

Gating Strategy:

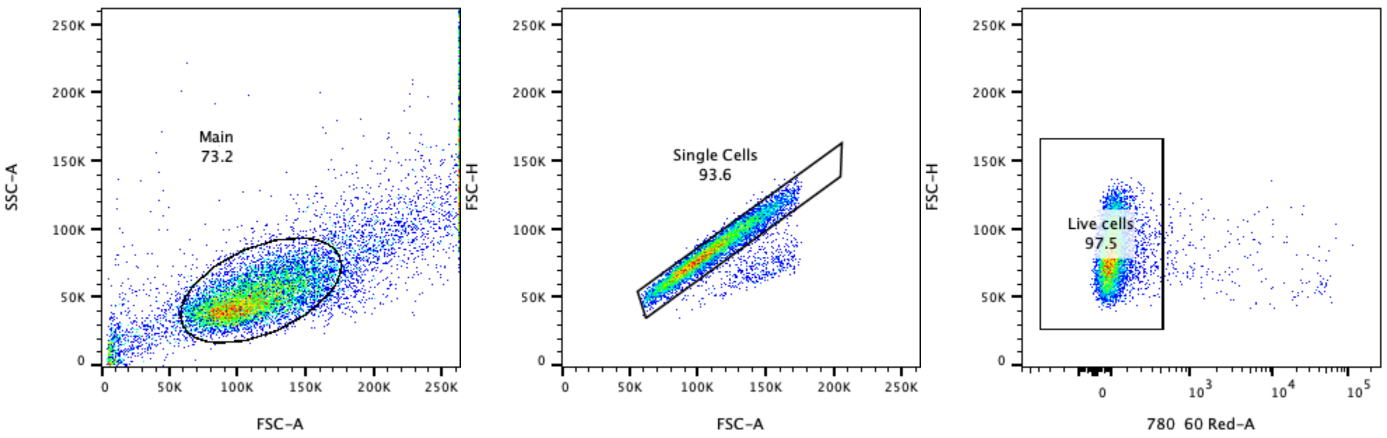

Representative dot plots:

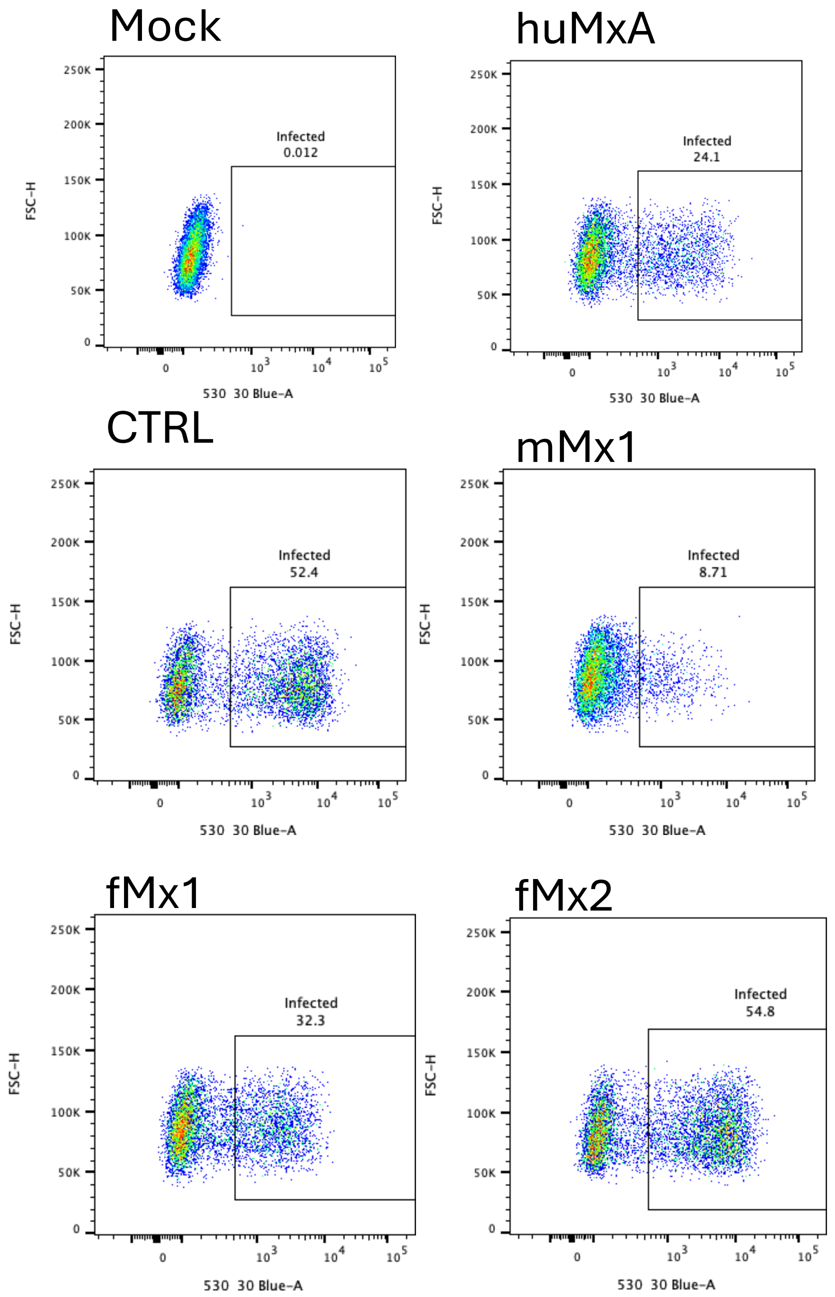

Supplementary data 4. Gating strategy and representative dot plots for flow cytometry data shown in Fig. 8A.
